# A convenient strategy to clone small RNA and mRNA for high-throughput sequencing

**DOI:** 10.1261/rna.071605.119

**Published:** 2020-02

**Authors:** Lichao Li, Hui Dai, An-phong Nguyen, Weifeng Gu

**Affiliations:** Department of Molecular, Cell, and Systems Biology, University of California, Riverside, California 92521, USA

**Keywords:** small RNA cloning, mRNA cloning, high-throughput or next-generation sequencing, RNA modifications, constructing sequencing libraries without gel purification

## Abstract

High-throughput sequencing has become a standard tool for analyzing RNA and DNA. This method usually needs a cDNA/DNA library ligated with specific 5′ and 3′ linkers. Unlike mRNA, small RNA often contains modifications including 5′ cap or triphosphate and 2′-*O*-methyl, requiring additional processing steps before linker additions during cloning processes; due to low expression levels, it is difficult to clone small RNA with a small amount of total RNA. Here we present a new strategy to clone 5′ modified or unmodified small RNA in an all-liquid-based reaction carried out in a single PCR tube with as little as 20 ng total RNA. The 7-h cloning process only needs ∼1 h of labor. Moreover, this method can also clone mRNA, simplifying the need to prepare two cloning systems for small RNA and mRNA; the barcoded PCR primers are also compatible with non-cDNA cloning applications, including the preparation of genomic libraries. Not only is our method more convenient for cloning modified RNA than available methods, but it is also more sensitive, versatile, and cost-effective. Moreover, the all-liquid-based reaction can be performed in an automated manner.

## INTRODUCTION

High-throughput sequencing has become a revolutionary technique for analyzing DNA/RNA. As the cost has significantly decreased over the past decade, it is becoming a standard tool for gene analyses in biomedical fields. Therefore, any technical advance in this technique will have a broad impact. Since a single sequencing run is usually sufficient for analyzing multiple samples, individual samples are usually cloned with specific barcodes, pooled, and sequenced as a single library for cost-sharing, and then debarcoded to obtain sample-specific sequences (Stiller et al. 2009; Gu et al. 2011). Although this strategy significantly decreases the sequencing cost, it cannot solve the problem that the overall library construction cost using commercial kits could easily surpass the sequencing cost.

Usually DNA, mRNA, and small RNA sequencing libraries are constructed with distinct linkers and/or chemistry, forcing customers to purchase different kits (Reuter et al. 2015). Lack of compatibility among these expensive kits and transparency for the protocol details often lead to confusion and waste. Most commercial kits only provide just enough primers for a specified number of library constructions, allowing no mistakes or errors during cloning. Since the linkers for DNA, mRNA, and RNA libraries are different, the corresponding PCR primers for obtaining the final amplicons are also different. If a laboratory wants to prepare its own barcoded PCR primers, it has to prepare three sets.

In most sequencing platforms, a DNA or cDNA library is made by ligating fragmented DNA/RNA with platform-specific linkers, which are then used to design primers for amplifying and reading sequences/barcodes (Goodwin et al. 2016). The DNA library construction strategy is straightforward, basically ligating fragmented-and-end-fixed target DNA with partially double-stranded DNA linkers, as used by commercial kits (Illumina 2012; Kircher et al. 2012). To clone RNA, RNA is converted to cDNA with 5′ and 3′ linkers added for cDNA amplification and sequencing. mRNA can be fragmented and then used to make cDNA with 5′ and 3′ linkers added using various methods including ligation and reverse transcription (RT); small RNA is usually first ligated with 5′ and 3′ linkers and then converted to cDNA. In the ligation-based methods, the 5′ ligation needs a 5′ monophosphate (p) and the 3′ ligation needs a 3′OH on target RNA. However, many small RNA species contain modifications at the ends (Chen 2005; Ruby et al. 2006; Kirino and Mourelatos 2007; Pak and Fire 2007; Batista et al. 2008; Affymetrix ENCODE Transcriptome Project and Cold Spring Harbor Laboratory ENCODE Transcriptome Project 2009; Taft et al. 2009; Conine et al. 2010; Gu et al. 2012). For example, *Caenorhabditis elegans* 22G-RNA (22G) bears a 5′ triphosphate, which is incompatible with 5′ ligation (Pak and Fire 2007; Gu et al. 2009); capped small RNA or promoter-associated small RNA has a 5′ cap, which is also incompatible with 5′ ligation (Affymetrix ENCODE Transcriptome Project and Cold Spring Harbor Laboratory ENCODE Transcriptome Project 2009; Taft et al. 2009; Gu et al. 2012); and the 2′-*O*-methyl group on the last ribose significantly decreases the 3′ ligation efficiency under normal conditions (Gu et al. 2009; Munafó and Robb 2010). Strategies including enzymatic treatments have been developed to remove these modifications or overcome the inhibitory effects. Since commercial library construction kits usually do not provide such functionalities, additional treatment and purification steps are required. Without these additional steps, the experimental manipulation (actual human labor) may only take 1 h; however, with them, the manipulation time could be easily doubled, tripled or even more depending on the sample number.

Some small RNA cloning strategies adopt a bypass mechanism to avoid a direct 5′ RNA ligation. For example, after 3′ ligation, RNA is converted to cDNA and then cDNA is ligated with a 3′ linker, which corresponds to a 5′ linker ligated to RNA (Pak and Fire 2007). A second bypass mechanism uses cDNA circularization and then the 3′ linker is cut into two parts: one flanking the 5′ end and the other flanking the 3′ end of insert cDNA (Kwon 2011). The third bypass mechanism utilizes the template switch activity of reverse transcriptases to add a 3′ linker to cDNA, which corresponds to a 5′ linker ligated to RNA (Ko and Lee 2006). These bypass mechanisms can overcome the ligation difficulty associated with the RNA 5′ modifications since the corresponding 3′ end of cDNA does not have any modification. However, the 5′ ligation of RNA usually serves as a selection for 5′p-RNA including miRNA, Dicer-dependent siRNA, and piRNA. Without it the final library may contain a significant fraction of cDNA derived from degraded RNA, which usually bears 5′OH. Not only does this contamination reduce the cloning yield of authentic RNA, but it also generates artifacts, leading to wrong conclusions. Moreover, bypass mechanisms equalize RNA 5′ ends, meaning that specific modification information is lost. Therefore, these methods cannot be used to only clone or enrich a specific group of 5′ modified RNA under normal conditions.

We have developed a unified strategy for constructing high-throughput small RNA/mRNA libraries. Since the linkers are derived from a strategy for making high-throughput DNA libraries, the barcoded PCR primers here can be used to amplify high-throughput DNA libraries too. Our strategy is capable of cloning modified and unmodified small RNAs using the enzymatic treatments including dephophorylating RNA by *C. elegans* PIR-1 and decapping RNA by human Dcp2 (hDcp2). Unlike the previously reported methods, these treatments are coperformed in the linker ligation reactions, avoiding the steps for enzyme removal/inactivation and/or buffer exchange. All the steps are performed inside a PCR tube in an all-liquid-based manner, significantly reducing labor time. This method is able to construct a small RNA library using as little as ∼20 ng of total RNA, a level much lower than the amount required by available commercial kits. Since it is all-liquid-based, it can be adapted to automation. The cost is minimal since the method only needs a few common enzymes, which can be purchased or easily purified using a single His-tag purification. In all, our strategy is more sensitive, convenient, versatile and cost-effective than most available methods.

## RESULTS

### Designing linkers and primers for cloning small RNA and mRNA based on the DNA cloning system

**FIGURE 1.**
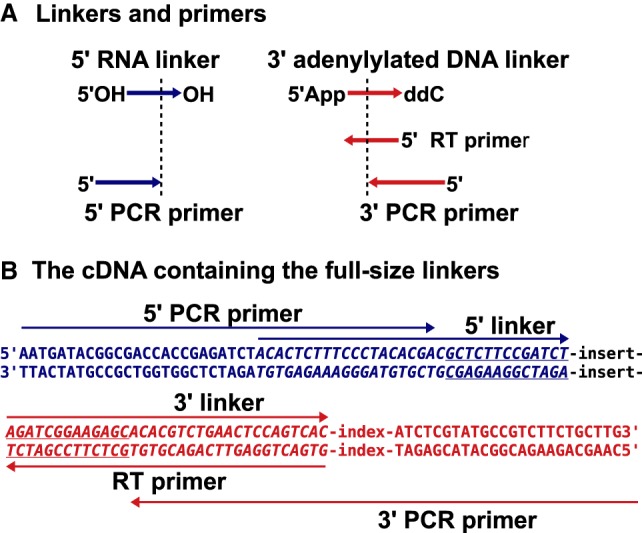
The linker and primer design. (*A*) The linkers and primers: The 5′ linker is an RNA oligo with 5′OH and 3′OH; the 3′ linker is an adenylylated DNA oligo with 5′App and 3′ ddC (dideoxyC); the RT primer is reverse complementary to the 3′ linker; and the 5′ and 3′ PCR primers partially overlap with the 5′ and 3′ linkers, respectively. (*B*) The sequence feature of the final cDNA: The amplicon is drawn as a double-stranded DNA with five arrows indicating the primer/linker sequences; the underlined parts are the 13-nt inverted repeats flanking the inserts; and the index represents an 8-nt barcode.

Different linkers are used for cloning small RNA, mRNA, and DNA due to distinct reaction mechanisms. We aimed to use the same linker system to clone all of them so that we can amplify the final DNA/cDNA amplicon using the same PCR primers including 64 barcoded 3′ primers and one 5′ primer. Our design is based on the Illumina platform simply due to its popularity. For DNA cloning, the last 13 nucleotides (nt) of the 5′ linker and the first 13 nt of the 3′ linker ligated to each target DNA strand form double-stranded DNA, a commonly used strategy required for DNA-based ligation (Fig. 1; Illumina 2012). Since we desired a unified linker system, our linkers for RNA cloning were designed based on a DNA cloning system. However, the 13-nt sequences are not used for designing PCR primer simply to avoid primer specificity issues. The 5′ linker *5*′*OH-ACACUCUUUCCCUACACGACGCUCUUCCGAUCU-OH* is an RNA oligo with 5′OH blocking the RNA ligase-mediated activation (adenylylation with ATP) (Fig. 1). Therefore, this linker can only serve as a ligation acceptor using the 3′OH but not a ligation donor with the 5′ OH. In contrast, the 3′ linker *5*′*App-AGATCGGAAGAGCACACGTCTGAACTCCAGTCAddC* is an activated DNA oligo (adenylylated) which allows for ligation without ATP, that is, serving as a ligation donor with the 3′ ddC (dideoxyC) blocking its usage as a ligation acceptor (Fig. 1). This design determines the ligation direction, that is, the 3′ linker to the target and then the 3′ linker-target to the 5′ linker. These linkers are further extended to contain the full-size linkers in PCR reactions using 5′AATGATACGGCGACCACCGAGATCT*ACACTCTTTCCCTACACGA* and 5′CAAGCAGAAG ACGGCATACGAGAT-NNNNNNNN-*GTGACTGGAGTTCAGACGTGT*, in which N represents an 8 nt barcode and the italic parts represent the sequences derived from the ligation linkers (Fig. 1B).

A complete list of the ligation linkers, RT primer, and PCR primers including 64 barcoded 3′ primers are provided in the Supplemental Material (Supplemental Table S1).

### Constructing a small RNA high-throughput sequencing library

**FIGURE 2.**
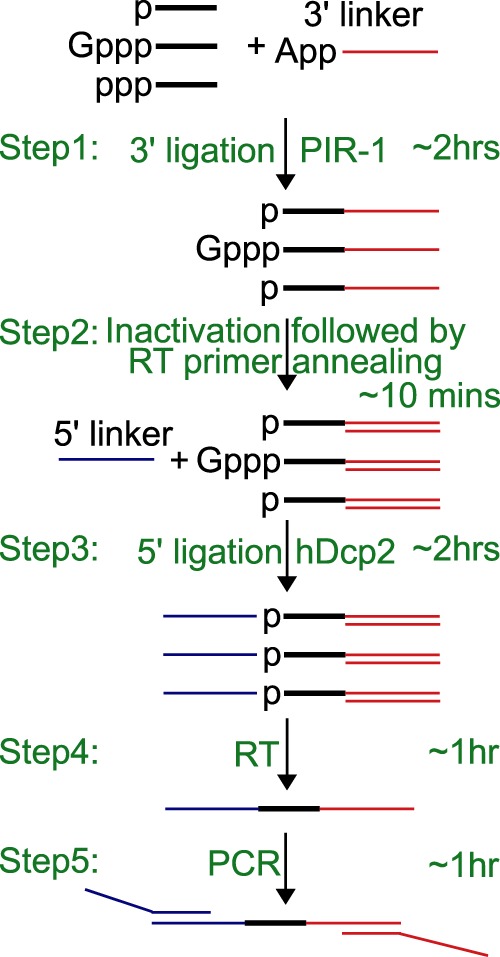
Strategy to make a small RNA library. RNA is ligated to an adenylylated 3′ linker using the truncated RNA ligase 2 while 5′ ppp-RNA is dephosphorylated with PIR-1 at step 1; the reaction is inactivated at 65°C for 10 min and an RT primer is annealed to the 3′ linker at 65°C for 5 min at step 2; a 5′ linker is ligated with the target RNA using T4 RNA ligase 1, while hDCP2 is added to decap capped RNA at step 3; an RT is performed to obtain the first strand cDNA at step 4; and cDNA is amplified and extended to obtain full-size 5′ and 3′ linkers at step 5.

We first developed a protocol to clone small RNAs. As shown in Figure 2 and Supplemental Table S2, small RNA is first ligated with 0.5 µM activated 3′ linker using 0.5 µM truncated T4 RNA ligase 2 in 10 µL buffer containing 50 mM Tris (pH 7.5), 10 mM DTT, and 10 mM MgCl_2_. Since the 3′ linker is 3′ ddC-modified primarily for blocking 3′ linker self-ligation, it can only be ligated to the 3′OH of target RNA. This strategy also avoids the activation of the 5′ phosphate of target RNA since (i) no ATP is used and (ii) the truncated T4 RNA ligase 2 does not possess the adenylylation activity (Ho and Shuman 2002; Nandakumar et al. 2004; Aravin and Tuschl 2005). To clone the 3′-end 2′-*O*-methylated RNA including piRNA and some miRNA, 25% PEG-8000 is added (Munafó and Robb 2010). T4 RNA ligase 1 can substitute for the truncated T4 RNA ligase 2 in the same reaction condition (Gu et al. 2011). However, T4 RNA ligase 1 can activate (adenylylate) target RNA even without addition of ATP likely because: (i) At least a fraction of T4 RNA ligase 1 is bound with ATP or adenylylated; and (ii) T4 RNA ligase 1 transfers AMP from the activated 3′ linker to adenylylate target RNA (Gu et al. 2011). This activation may cause target RNA circularization or target RNA–RNA ligation, decreasing the yield of target RNA-linker ligation. However, by controlling the amount of T4 RNA ligase 1 and ligation time, a satisfactory result could be easily achieved (Gu et al. 2009). T4 RNA ligase 1 does have an advantage since it can ligate 2′-*O*-methylated RNA more efficiently than the truncated T4 RNA ligase 2 (Gu et al. 2011, 2012).

After the 2-h 3′ ligation, the reaction is first heat-inactivated for (i) denaturing the truncated T4 RNA ligase 2; and (ii) annealing with ∼0.5 µM RT oligo to the 3′ linker either ligated or unligated by adding 0.5 µL of a 10 µM RT oligo. And then 0.4 µM 5′ linker, 0.5 mM ATP, 0.25 µM RNA ligase 1, and water are added to the reaction reaching a final volume of 20 µL for the 5′ ligation. T4 RNA ligase 1 activates the 3′-ligated target RNA if it contains or has been enzymatically treated to expose 5′ monophosphate (5′p). In contrast, the 5′ linker lacking 5′p cannot be activated. Therefore, the 5′ linker can only serve as a ligation acceptor ligated to the 3′-ligated and 5′-activated target RNA. The annealing of the RT oligo with the 3′ linker may reduce the ligation of the free 3′ linker, if any are left after the 3′ ligation, with the 5′ linker, since T4 RNA ligase 1 prefers single-stranded substrates. This strategy may significantly reduce the formation of 5′ linker-3′ linker ligation especially when the RNA substrate is much less than the 3′ linker, generating excessive 3′ linker, as discussed below in the single worm RNA cloning.

A 30-minute RT step follows the 5′ ligation with addition of ∼0.2 µM Superscript II or III (Invitrogen), ∼4 mM additional DTT, ∼0.4 mM dNTP, and ∼2 µL 10× RT dilution buffer (0.25 M Tris pH 8.8 and 0.75 M KCl) reaching a final volume of ∼24 µL. Then the enzymes are inactivated at 85°C for 5 min. The obtained cDNA is amplified and extended to obtain the full-size linkers by PCR. 8-nt barcodes are inserted in the 3′ PCR primers for labeling individual samples. A typical 50 µL PCR reaction is composed of 1× PFU buffer, 15 mM tetramethylammonium chloride for reducing primer dimmer, 0.1 mM dNTPs, 0.1 µM 5′ and 3′ primers, 5 µL RT reaction, and 1× PFU polymerase. The PCR is first amplified for 5 cycles (94°C 20 sec; 53°C 20 sec; 68°C 30 sec) and then amplified for 11 cycles (94°C 20 sec; 68°C 40 sec). Additional 0.6 µM 5′ and 3′ primers are added and the PCR is amplified for 2 more cycles (94°C 20 sec; 68°C 40 sec).

In all, the whole cloning process is performed in a single PCR tube with liquid components added sequentially and can be easily finished within ∼7 h including ∼ 1-h labor time. A formulated working protocol is presented in the Supplemental Table S2.

### Achieving high reliability and sensitivity

**FIGURE 3.**
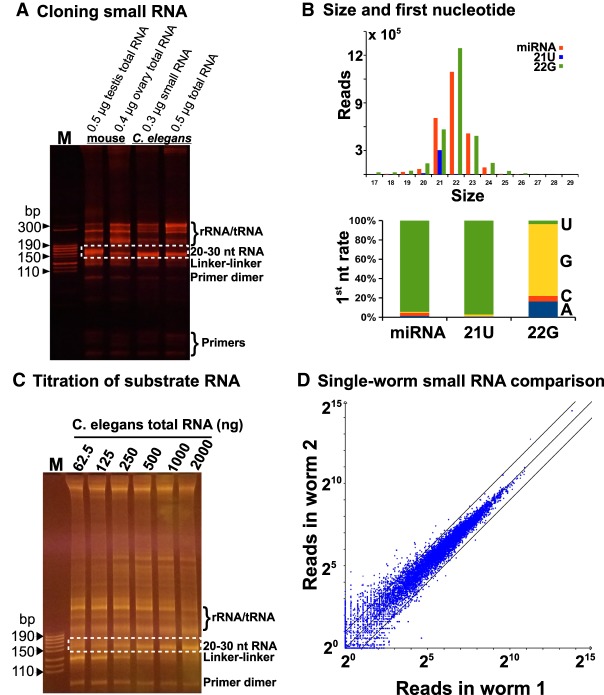
Analysis of sensitivity and reliability of the cloning strategy. (*A*) 0.5 µg mouse testis total RNA, 0.4 µg mouse ovary total RNA, 0.3 µg *C. elegans* small RNA of size less than 200 nt, and 0.5 µg *C. elegans* total RNA were used to clone small RNA, and 5 µL of each PCR product was resolved on an 8% native PAGE gel. The inserted RNA in the PCR product is labeled on the *right* as well as the linker–linker ligation product, primer dimers, and free primers, as compared to the DNA size marker (M); the dotted box represents the 20–30 nt RNA inserts. (*B*) The size and first nucleotide analyses of miRNA, 21U, and 22G cloned using 0.5 µg *C. elegans* total RNA treated with PIR-1. (*C*) A twofold serial titration of *C. elegans* total RNA (62.5–2000 ng) was used to examine the cloning sensitivity threshold with the dot-boxed area representing the desired amplicon and “M” representing the DNA size marker. (*D*) The comparison of 22G derived from two single worms with each blue dot representing one gene, with “X” mapped to 22G reads in worm 1 and “Y” reads in worm 2.

Our ultimate goal is to use the above strategy to clone small RNA including miRNA, piRNA and siRNA, and fragmented mRNA. Since fragmented mRNA is basically small RNA, we only optimized the conditions to achieve high reliability and sensitivity using in vivo small RNA, and then applied these conditions to clone fragmented mRNA. We first examined our method by cloning small RNA using total RNA isolated from mouse testes, mouse ovaries, and *C. elegans* adult worms*,* and using purified *C. elegans* small RNA of size less than 200 nt, which contain miRNA, 22G-RNA (siRNA), 21U-RNA (piRNA), tRNA, and 5.8S/5S rRNA (Ruby et al. 2006; Gu et al. 2009). As expected (Fig. 3A), all the samples generated a ∼150-basepair (bp) cDNA band which contains both the linker and the RNA insert. The size of the band in the testis sample is ∼5 bp bigger than the bands in other samples since the major RNA species in the testes is the 25–30 nt piRNA while other samples contain the 20–23 nt small RNA (Ruby et al. 2006; Kirino and Mourelatos 2007; Gu et al. 2009; Tushir et al. 2009). We sequenced the library constructed from 0.5 µg *C. elegans* total RNA, and found that 94% of the cDNA was derived from authentic small RNA including miRNA, siRNA, and piRNA. The size distribution and first nucleotide preference of each small RNA species are the same as reported (Gu et al. 2009). For example, miRNA peaks at ∼22 nt and prefers 5′ U; 22G-RNA peaks at 22 nt and prefers 5′ G; and 21U-RNA peaks at 21 nt and prefers 5′ U (Fig. 3B).

The above experiment indicates that our method is very sensitive since we were able to make a small RNA library using 0.4 µg mouse ovary total RNA, which contains the least small RNA in all the samples (Fig. 3A). To further determine the sensitivity threshold, we used a twofold titration of *C. elegans* total RNA ranging from 62.5 to 2000 ng as the substrate and observed a clear cDNA band with small RNA inserts from all the samples (Fig. 3C). We also found that the method worked with 31 and 16 ng total RNA in a separate titration experiment (data not shown). All these experiments were performed using our standard condition in a single-tube-liquid-based manner.

To examine the reliability of our method especially when using a tiny amount of total RNA, we decided to establish a complete protocol starting from isolating total RNA from single worms, cloning it and using bioinformatics to compare the results. A single worm contains 10–20 ng total RNA from ∼2000 cells, as estimated from the total RNA yield extracted from ∼10,000 adult worms. This amount is equal to the total RNA derived from ∼500–1000 mammalian cells. Based on the intensity of the ∼22 nt small RNA band stained with Ethidium Bromide or SyBr Gold, we estimated that there is ∼1 ng small RNA per 10 µg total RNA in adult worms. We isolated total RNA from single worms using proteinase K digestion and used it to clone the ∼22 nt small RNA, which was ∼1.5 pg or 0.21 fmole, as estimated using the above parameters. Given it is unlikely to obtain a 100% yield for single-worm RNA isolations, the actual amount of RNA used for cloning should be <0.21 fmole. For such a tiny amount of starting material, we decided to use half enzymes and linkers in the 3′/5′ ligation and RT steps as compared to our standard procedure, minimizing the formation of linker-linker (no RNA insert) or other byproducts. The result clearly indicates that our method is able to clone small RNA at fmole level from single worms and deliver high reliability at this level, as the two individual worm samples exhibited consistent small RNA profiles (Fig. 3D).

### Cloning 5′ triphosphorylated RNA (ppp-RNA)

**FIGURE 4.**
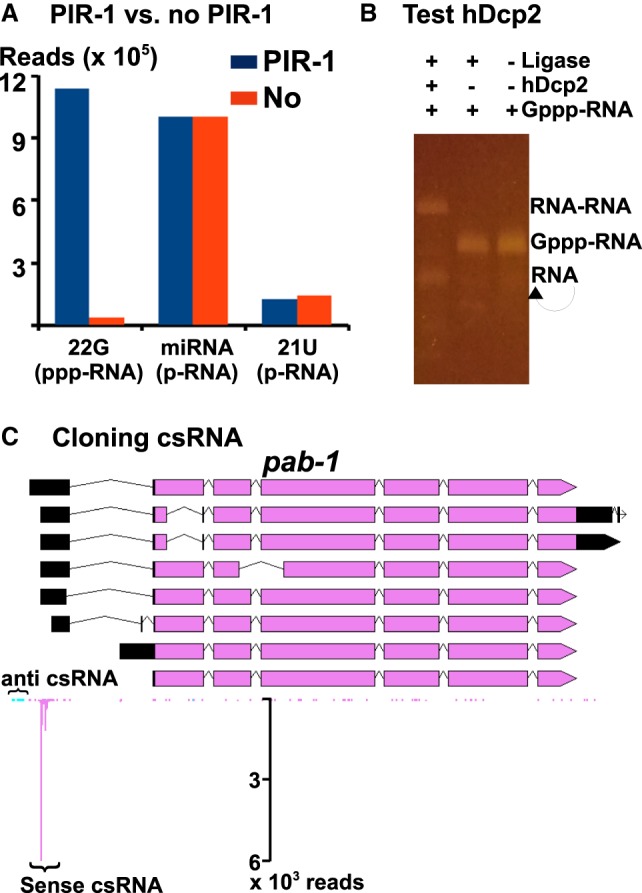
Cloning of ppp-RNA and csRNA. (*A*) The comparison of small RNAs cloned with PIR-1 or no PIR-1, as normalized to the total miRNA, which is equally cloned in both methods. (*B*) A capped RNA substrate is self-ligated (RNA–RNA) or circularized (a single RNA alone) by T4 RNA ligase 1 with hDcp2. (*C*) Cloned csRNAs within the *pap-1* promoter region with “pink” representing sense reads and “blue” representing anti-sense reads.

Small ppp-RNA cannot be directly ligated at the 5′ end. To clone it, ppp-RNA is usually treated with commercial RNA polyphosphatases, generating 5′ monophosphorylated RNA (p-RNA), which is compatible with 5′ ligation (Gu et al. 2009). However, the available enzymes need special buffers and temperature incompatible with RNA ligation. Therefore, ppp-RNA is usually pretreated enzymatically for dephosphorylation, extracted with organic reagents for enzyme removal, and precipitated for buffer exchange. This process is tedious, time-consuming, and counterproductive for cloning efficiency (Gu et al. 2009). If we have adopted this strategy, the labor time for the cloning procedure would have doubled and the cloning efficiency would have decreased due to sample loss. We aimed to utilize an RNA polyphosphatase which works efficiently in the ligation condition. Previous studies have shown that human PIR1 dephosphorylates ppp-RNA, generating p-RNA. However, it works at 37°C, a temperature incompatible with the ligation condition. Since PIR-1 is highly conserved and *C. elegans* PIR-1 works at 20°C, it is a perfect candidate for the desired activity. We obtained a recombinant *C. elegans* PIR-1 of homogeneity from *E. coli* (Supplemental Fig. S1). To examine its dephosphorylation activity, we coapplied this enzyme in the 3′ ligation step with the truncated T4 RNA ligase 2, generating 3′ ligated and 5′ p-RNA using the same reaction. Then the RNA was further 5′ ligated. The high-throughput sequencing analysis confirmed that this strategy worked efficiently for cloning ppp-RNA. As shown in Figure 4A, p-RNA including miRNA and 21U-RNA was cloned efficiently with and without PIR-1. In contrast, 22G-RNA (ppp-RNA) was only efficiently cloned with PIR-1 (Fig. 4A). The 22G proportion in the PIR-1 treated sample was ∼ 50%, a ratio very close to the one reported previously using the samples pretreated with Tobacco Acid Pyrophosphatase (Gu et al. 2009).

### Cloning capped small RNA

Capped small RNA (csRNA) or promoter-associated small RNA is generated during transcription initiation and bears a 5′ G cap with size <200 nt (Affymetrix ENCODE Transcriptome Project and Cold Spring Harbor Laboratory ENCODE Transcriptome Project 2009; Taft et al. 2009; Gu et al. 2012). The cap structure is incompatible with 5′ ligation by RNA ligases. Tobacco acid pyrophosphase (TAP) had been routinely used to decap csRNA, generating 5′ p-RNA, before the manufacturer discontinued this enzyme (Gu et al. 2012). Several alternative decapping enzymes have been developed including Edc1-fused Dcp1–Dcp2 (Paquette et al. 2018). Our goal is to find a decapping enzyme compatible with the ligation condition. We obtained a hDcp2 construct from Dr. Megerditch Kiledjian (Wang et al. 2002), purified a recombinant hDcp2 expressed from *E. coli* and examined its decapping activity in the ligation buffer. hDcp2 worked well in the 5′ ligation step with T4 RNA ligase 1 (Fig. 4B), as the capped RNA substrate cannot be ligated (RNA–RNA in Fig. 4B) or circularized with the ligase but became ligatable with the hDcp2 treatment. We then used hDcp2 to construct a csRNA library. Since csRNA is not as abundant as other small RNA species, we first dephosphorylated the abundant small RNA species using Calf Intestinal Phosphatase (CIP), making them 5′ unligatable with T4 RNA ligase 1, and then applied hDcp2 in the 5′ ligation reaction to decap csRNA, making it 5′ ligatable. As shown in Figure 4C, both sense and anti-sense (anti) csRNAs around the promoter area of *pab-1* were cloned and they were separated by ∼150 nt, a typical distance as reported (Affymetrix ENCODE Transcriptome Project and Cold Spring Harbor Laboratory ENCODE Transcriptome Project 2009; Taft et al. 2009; Gu et al. 2012).

In our condition, hDcp2 does not work well when added in the 3′ ligation step (data not shown). Since the buffer conditions for the 3′ and 5′ ligations are almost the same, we suspect that the hDcp2 we purified may be contaminated with a low level of RNA phosphatases, which dephosphorylated the p-RNA generated by hDcp2 in the 2-h 3′ ligation step, making it incompatible for the following 5′ ligation. In contrast, when hDcp2 is added in the 5′ ligation, the ligase may add a linker to the 5′p of the decapped RNA before dephosphorylation by any RNA phosphatase.

### Cloning mRNA

**FIGURE 5.**
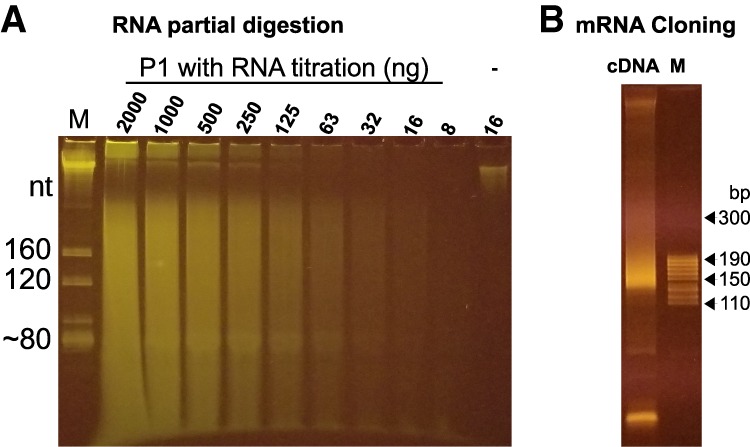
Cloning mRNA. (*A*) Partial digestion of RNA using nuclease P1. M, a total RNA size marker with the estimated sizes using 5.8S rRNA (∼160 nt), 5S rRNA (∼120 nt), and tRNA (∼80 nt); a twofold serial titration of substrate total RNA from 2000 to 8 ng was partially digested with nuclease P1; “-” is the negative control using ∼16 ng purified mRNAs incubated without P1. (*B*) Fragmented mRNA was cloned as a cDNA library and resolved on an 8% native PAGE gel, as compared to the size marker M.

In addition to cloning small RNA, we aimed to use the same method to clone mRNA. In general for mRNA cloning, mRNA is fragmented to small RNA, ligated with linkers, and converted to cDNA. However, a partial digestion of mRNA either chemically or enzymatically usually generates small RNA with 5′OH and 2′p or 3′p or cyclic phosphate at the 3′ end. For ligation-based cloning methods, these ends have to be converted to 5′p and 3′OH. To avoid these conversion steps, we utilized nuclease P1, an enzyme cutting single-stranded RNA or DNA, generating small RNA or DNA with 5′p and 3′OH (Fujimoto et al. 1974). A potential challenge for enzyme-mediated partial digestion is how to control over/under-digestion when RNA/enzyme ratios vary. It is very inconvenient to figure out a specific condition for each sample especially when sample concentrations are not quantifiable. By diluting the enzyme and examining different buffers, we eventually obtained a condition under which the substrate RNA amount from 8 to 2000 ng did not affect the size of fragmented RNA with a given amount of enzyme (Fig. 5A). We speculate that this condition was achieved because of an extremely quick interaction (digestion) between P1 and substrate RNA, meaning P1 almost behaves like a free-moving enzyme which, regardless of substrate RNA concentrations, crosses any given unit of distance at the same frequency. To minimize the effect of secondary RNA structures on digestion, the reaction is performed at 60°C, a temperature at which nuclease P1 works very efficiently. A neutral pH buffer is adopted to minimize RNA degradation by hydrolysis at 60°C. In all, we have established an RNA fragmentation method which is substrate-concentration-independent, insensitive to RNA secondary structures, and ligation-compatible. Moreover, this method is very quick, taking 10 min, very convenient, requiring one enzyme and a simple buffer, and inexpensive, costing a few cents per reaction.

We then constructed a library with poly (A)-containing mRNA fragmented using nuclease P1. The obtained mRNA profile is similar to that of a previous one made using alkali hydrolysis. For example, the reads spread randomly along the length of mRNA (Supplemental Fig. S2A) and the individual gene expression levels correlate well between the two samples (Supplemental Fig. S2B). Moreover, our method was capable of cloning at least 1 ng mRNA (data not shown), a sensitivity level sufficient for most routine work.

### Minimizing bulged PCR products to reduce amplification bias caused by PCR overcycles

**FIGURE 6.**
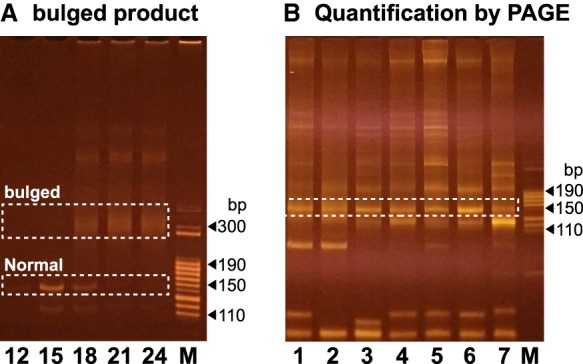
Quantification using a native PAGE gel. (*A*) PCR reactions produced perfectly based-paired normal (dotted box) products at low cycle numbers (12 and 15) and then bulged PCR products (dotted box) when the reactions were overcycled (18, 21, and 24). (*B*) Quantification using a PAGE gel: 5 µL PCR product was resolved using an 8% native PAGE gel. Dot-boxes are the small RNA amplicons for samples 1–7; M, the size marker.

For any given amount of template DNA, it is difficult to predict how many PCR cycles are needed to generate enough products with a minimal amount of undesirable byproducts such as primer dimers. In laboratory routines, PCR reactions are usually empirically overcycled to maximize yields. Under such conditions, primers are used up first and then product DNA is denatured and renatured without amplification, resulting in futile cycles. This may not cause any observable effect on PCR specificity or even yields, since PCR conditions are usually so stringent that only one product is generated per reaction, and thus denaturing/renaturing processes in overcycled PCR reactions do not change the product. However, overcycle may cause serious issues for constructing a library containing more than one product. For example, since different insert DNA/cDNA molecules are flanked by the same 5′ and 3′ linkers in high-throughput sequencing libraries, linker regions anneal to each other intramolecularly and/or intermolecularly once PCR reactions are overcycled. However, insert DNA/cDNA strands may not find their complementary strands, resulting in bulged products, that is, molecules with perfectly base-paired linkers flanking two single-stranded or partially annealed insert strands. Bulged products appear as smears running much slower on native PAGE gels than perfectly matched products since they contain inserts with different DNA compositions and secondary structures (Fig. 6A). Depending on overcycle number, bulged products could extend to a broad size range, leading to more purification work and reduced yield.

Bulged products are biased for less abundant DNA. In a PCR reaction generating only two products, AA and BB of the same size, three products AA, AB, and BB are generated by overcycling. If AA is 99% of AA + BB and BB is 1% before overcycling, AA, AB and BB are 98.01%, 1.98%, and 0.01%, respectively, after overcycling. In the bulged products, both the A and B compositions are 50%, while in the perfectly matched products, AA is ∼99.99% (98%/[98%+0.01%]) and BB is ∼0.01% (0.01%/[98%+0.01%]). Therefore, if perfectly matched products are selected, abundant DNA species are overrepresented, and if bulged products are selected, less abundant DNA species are overrepresented.

To solve the overcycle issue, theoretically both perfectly matched and bulged products can be purified together. However, PCR products often contain primer dimers and undesirable products, which form bulged products with desirable PCR products once PCR reactions are overcycled, basically making this purifying-all strategy ineffective. To solve this issue, we used to examine the PCR product at cycle number 12, 16, 20, and 24 from the same PCR reaction on a PAGE gel, obtain the maximal PCR number without overcycle, and use this number to mass produce the product (Gu et al. 2011). Although this solution worked well, it was tedious and the PCR reactions were usually overcycled at cycle 24. So we had to run another PCR reaction and gel purification to obtain the nonovercycled product. We have developed a new strategy to solve the overcycle problem with much less workload. We first amplify cDNA using 0.1 µM primers for 16 cycles and then add 0.6 µM primers for 2 more cycles. If a PCR reaction is overcycled at cycle 16, the two additional cycles convert it back to a nonovercycled reaction; if a reaction is not overcycled, the two more cycles cannot make it overcycled since the added 0.6 µM primers are excessive.

### A convenient quantification and pooled purification strategy

A high-throughput sequencing library usually contains multiple barcoded samples for cost-sharing. To obtain a specific composition, individual samples are purified, quantified and then mixed as a pooled sample. Since samples may have primer dimers and/or other byproducts including cDNA derived from rRNA and tRNA, a gel purification is required for obtaining target cDNA of specific sizes. This process is tedious and time consuming (Gu et al. 2011). To reduce workload, we developed a two-step method: (i) visually comparing the relative concentration of target DNA of specific sizes, say 140–170 bp containing miRNA, siRNA, and piRNA; (ii) pooling samples according to a desired ratio and gel-purifying the pooled sample (Fig. 6B). This way we were able to easily make a library consisting of 60 samples within 8 h. Although the visual quantification may not be perfect, we found that the variation was usually within threefold.

## DISCUSSION

Our major goal is to design a simple, convenient and cost-effective method for cloning small RNA. For this purpose, we have developed an all-liquid-based multistep reaction in a single PCR tube. The whole procedure takes ∼ 7 h with only ∼ 1 h labor time and the rest primarily used for increasing ligation efficiency of modified RNA. It can be shortened to ∼4 h for cloning unmodified miRNA (0.5 and 1 h for the 3′ and 5′ ligation, respectively, 30 min for RT and 1 h for PCR). Like the commercial kits, our method starts with total RNA, a strategy providing convenience but generating byproducts derived from tRNA and rRNA. In addition, the whole procedure involves no gel purification or DNA precipitation (the pooled DNA amplicons are still gel-purified, as discussed below). Our method is much more sensitive than the commercial kits, working with as little as 16 ng total RNA. We speculate that we could start with even less total RNA if using one gel purification to enrich target cDNA amplicons while removing primer dimmers and linker-linker ligation byproducts, followed by a second round of PCR amplification for mass producing target cDNA amplicons.

We prefer ligation reactions for adding 5′ linkers to target RNA over other techniques, for example, cDNA circularization or template switching via reverse transcriptases. Since most degraded RNA bears a 5′OH, it cannot be cloned using ligation, which requires a 5′p. Therefore, not only does the 5′ ligation add a linker for cDNA application and sequencing, but also serves as a selection mechanism for enriching authentic small RNAs. Due to this selection, our method works well with samples heavily contaminated with degraded RNA including some immunoprecipitated RNA samples. At the same time, our method can be easily adapted to clone RNA bearing a 5′OH simply by adding T4 Polynucleotide Kinase (PNK) in the 5′ ligation step (data not shown).

Our method only needs a few common enzymes such as T4 RNA ligases, reverses transcriptases, DNA polymerases and PIR-1 and hDcp2. The last two are required for processing 5′ modified RNA but not for p-RNA including miRNA, Dicer-dependent siRNA, and piRNA. We easily obtained RNase-free enzymes using a single His-tag-mediated purification. The first three enzymes above are also commercially available. However, for some reasons, when tested by other labs, the protocol did not work well with some commercial enzymes, but worked well with our purified enzymes. We believe that the failure may be caused by the commercial T4 RNA ligase since commercial SuperScript III and Taq worked in our method. We speculated that the commercial T4 RNA ligases used may be contaminated with a trace amount of RNases or phosphatases. We estimate that the cost per library construction is negligible if using home-made enzymes and activated 3′ linkers, and less than $10 if using commercial enzymes and 3′ activated linkers.

Our method clones all types of small RNA including 5′ and/or 3′ modified RNA. Commercial kits are usually optimized for cloning small RNA with 5′p and 3′OH. Additional enzymatic steps and conditions are needed for processing csRNA, 5′ppp-RNA, 5′OH-RNA and 3′-modified RNA (Gu et al. 2009, 2012). These steps could easily double labor time and lead to reduced cloning efficiency. In our method, all steps are performed in one single liquid-based reaction, significantly reducing workload. piRNA usually contains 2′-*O*-methyl at the 3′ end, which decreases the 3′ ligation efficiency. This inhibitory effect is overcome by addition of 25% PEG-8000 (Munafó and Robb 2010). PIR-1 is used to modify ppp-RNA in the 3′ ligation, generating p-RNA which is compatible with 5′ ligation. This is a convenient strategy for cloning *C. elegans* small RNA since ∼50% of it is ppp-RNA (22G-RNA) (Gu et al. 2009). csRNA is decapped into 5′ p-RNA by hDcp2 and ligated in the 5′ ligation step. However, csRNA is usually expressed at an extremely low level, decapping only makes them 5′ ligatable but not enriched (Gu et al. 2012). To enrich csRNA, 5′ p-RNA, usually the major small RNA species, is dephosphorylated, making it 5′ unligatable, and then csRNA is decapped for 5′ ligation (Gu et al. 2012). 5′ OH-RNA is cloned by addition of PNK in the 5′ ligation step, as discussed below for mRNA cloning. Theoretically, if all these enzymes are used, the method allows for cloning of all cellular small RNAs.

We aimed to develop a versatile method for cloning both mRNA and small RNA and to use the same sets of PCR primers to amplify libraries derived from DNA and RNA. Since the 5′ and 3′ linkers are derived from the DNA cloning system, the PCR primers definitely work with DNA libraries. Actually, we used these primers to amplify DNA libraries constructed using a commercial kit (data not shown) without purchasing the expensive barcoded primers. For mRNA cloning, we first used alkali-hydrolysis to fragment mRNA, generating small RNA with 5′OH and 3′ cyclic phosphate. Then we used PNK to fix the ends in the 3′ ligation step by: (i) removing the 3′ cyclic phosphate at room temperature for ∼5 h; and (ii) phosphorylating the 5′ end with addition of ATP at 37°C for 1 h (data not shown). Although it works well, the 3′ ligation step takes ∼6 h, making it impossible to finish the cloning process within 1 d. This prompts us to use nuclease P1, which generates ligation-compatible ends. The cost of nuclease P1 is close to alkali-hydrolysis, basically nothing. Nuclease P1 works well at 60°C, significantly reducing the effect of RNA secondary structures on the digestion pattern and efficiency. Moreover, our partial digestion condition is insensitive to the RNA amount so that there is no need to optimize the enzyme/RNA ratio for getting fragmented RNA of desired size.

All in all, our method is convenient, sensitive, and versatile. Any laboratory with basic molecular techniques can establish an integrated system to clone small RNA and mRNA.

## MATERIALS AND METHODS

### Total RNA and single-worm RNA preparation

RNA was extracted using TRI reagents (Sigma T9424) with a tissue tearor according to the manufacturer's protocol. Worms were grown on OP50 at 20°C for ∼70 h. To prepare single worm RNA, individual worms were transferred to a PCR tube and incubated with 0.4 µg/µL proteinase K in 10 µL buffer containing 40 mM Tris (pH 7.5), 10 mM EDTA, 0.3 M NaCl, 0.5% NP-40 and 0.5% SDS at 65°C for 10 min. The resulting RNA was phenol/chloroform extracted and coprecipitated with 20 µg glycogen.

### Purifying a recombinant *C. elegans* PIR-1 protein

The coding sequence of *C. elegans* PIR-1 gene, T23G7.5a.1, was integrated into pET-28a between the NdeI and BamHI restriction sites. The resulting plasmid was transformed into the BL21 (DE3)-RIL strain for protein expression. To express the recombinant PIR-1, a single colony was grown in 5 ml Terrific Broth (TB) medium containing 50 µg/mL Kanamycin and 20 µg/mL chloramphenicol at 37°C for 8 h; the whole culture was inoculated into 1 L TB media containing 50 µg/mL Kanamycin and 20 µg/mL chloramphenicol to grow ∼24 h at room temperature, reaching OD_600_ 0.5; and the protein expression was induced using 0.5 mM IPTG at 16°C for 15 h. The cells were pelleted and resuspended in 25 mL lysis/wash buffer containing 50 mM Tris-HCl (pH 7.5), 0.5 M NaCl, 5 mM 2-mercaptoethanol, 5% glycerol, 10 mM imidazole, 0.01% NP40, and 1 mM PMSF. The cells were sonicated on ice using 30 cycles of 20-sec sonication followed by 40-sec pause. The resulting solution was centrifuged at 20,000*g* at 4°C for 10 min and the supernatant was mixed with 2.5 mL HisPur Ni-NTA (Thermo Fisher Scientific) beads, which were prewashed three times with the lysis/wash buffer without PMSF. After 1-h nutation at 4°C, the beads were washed with 200 mL lysis/wash buffer in a 20 mL column and the protein was eluted using 10 of 0.5 mL lysis/wash buffer containing 0.4 M imidazole. The fractions 5–7, which contained the majority of the protein, were pooled and loaded onto a HiPrep Sephacryl S-100 HR column and fractionated with the imidazole-free lysis/wash buffer using the NGC Quest 100 Plus Chromatography System (Bio-Rad). The FPLC fractions contained a recombinant PIR-1 of high homogeneity (Supplemental Fig. S1). The pooled fractions were dialyzed for storage at −80°C using a buffer containing 20 mM Tris (pH 7.5), 100 mM NaCl, 1 mM DTT, 0.01% NP-40, 0.1 mM EDTA, and 50% glycerol.

### Adenylylation of the 3′ linker

T4 DNA ligase was used to adenylylate a 3′ linker oligo 5′p-AGATCGGAAGAGCACACGTCTGAACTCCAGTCA/ddC/, which together with 5′ACGGCATACGAGGGAAG/ddC/ was annealed to 5′CTCTTCCGATCTGCTTCCCTCGT A/ddC/ at 95°C for 2 min followed by a slow cooling (0.1°C/sec) to room temperature, forming a nicked double-stranded DNA at 10 µM concentration in 10 mM Tris pH 7.5 and 30 mM KCl. Then 2.5 µM of the annealed DNA was incubated with 2.5 µM of T4 DNA ligase in 1× DNA ligation buffer at 37°C for 1 h, followed by phenol extraction, DNA precipitation and purification using a 15% PAGE/7M urea gel.

### Partial digestion of RNA using nuclease P1

A total of 8–2000 ng total RNA was partially digested with 0.01 unit of nuclease P1 in a buffer containing 50 mM sodium citrate (pH 7.0) and 10 mM MgCl_2_ at 60°C for 10 min, generating RNA fragments with a median size of ∼150 nt.

### Bioinformatical analysis

High-throughput sequences were analyzed using our previous custom PERL (5.10.1) scripts and Bowtie 0.12.7 (Langmead et al. 2009; Gu et al. 2012). For *C. elegans* analyses, reads were mapped to the genome (WormBase release WS215) and the Generic Genome Browser was used to visualize the alignments (Stein et al. 2002). The software package is stored at https://github.com/guweifengucr/Wglab_small_RNA_analysis; the high-throughput data GSE129664 is accessible using https://www.ncbi.nlm.nih.gov/geo/query/acc.cgi?acc=GSE129664 with token axebgasejniltox; and the genome browser track is accessible using http://wglabpred.dyn.ucr.edu/cgi-bin/gbrowse/wg120.

## SUPPLEMENTAL MATERIAL

Supplemental material is available for this article.
